# Contract Service by Railway Surgeons

**Published:** 1895-02

**Authors:** 


### CONTRACT SERVICE BY RAILWAY SURGEONS.

There is a growing tendency to regard railroad surgery as a specialty, and railroad surgeons as a privileged class in the medical profession. It may not prove unprofitable to look into the grounds upon which such claims are based.

In the outset it is proper to ascertain whether the nature of the violence inflicted in connection with railroads differs so materially from injuries produced by other causes as to place them in a distinct division of wounds or not. The extremes of destructive crushing of parts and the slightest abrasions may result from railroad accidents, and all the intermediate varieties of contusion and laceration of structures are met with, as consequences of the various forces brought to bear upon the members of the body in being exposed to such a multiplicity of injuries. Falls upon irregular surfaces in attempting to get on or off moving trains are frequent causes of grave injury; the loss of equilibrium when standing or walking in a car which is very suddenly stopped or started often precipitates the individual against solid bodies, as the seats or framework of the coach, and thus wounds of a serious nature are produced. But the most typical forms of violence are those resulting from collisions of running trains, when a most powerful shock is communicated to all the occupants of a car, and the crushing of the timbers leads to terrible mangling of persons by the broken fragments, or to the scalding of those who may be exposed to the steam escaping from a broken boiler. The more common, but not less fatal results of being caught between the bumpers of cars in coupling or being thrown under the wheels of a train in motion, are matters of almost daily observation.

The accidents from explosions connected with mining and the bursting of cannon in military operations are the ordinary results of violence, which correspond in their nature to destructive railway injuries; and yet there are many occasions connected with cyclones and the tumbling down of large buildings from other causes, which prove equally serious in the maiming of limbs and loss of life to the inmates. The general characteristics of such injuries differ more in degree than in kind, and the element of crushing and laceration of structures is most prominent with the profound impression upon the nerve centers.

There is an impression that most of these injuries have some peculiar feature of severity or aggravation, which does not pertain to the results of violence from other sources; and it has been generally held by railroad officials, that only those medical men who have a certain kind of training and familiarity with these accidents are competent to treat them. This view of the relations of surgeons to this class of cases has induced the authorities to select special medical attendants for their respective companies, who are called upon to render their services at stipulated rates below the standard of compensation recognized by the medical profession of the different places where they are located. Strange as it may seem, when such terms are refused by one medical practitioner of a section, they have no difficulty in finding some other who is willing to make a bargain of this kind for his services. There is a fundamental objection to this contract system in the violation of the code of ethics recognized by the profession.

But the railroad surgeon seems to have no regard for precedents, and manifests a disposition to form alliances with others of his class, thus cutting loose from association with other colleagues. They are not content with local and national associations, but it is now proposed to establish an exclusive body under the title of "The American Academy of Railway Surgeons," to "be confined to only the more eminent surgeons in the railway service." It is inferred that out of the two thousand or more connected with the National Association of Railway Surgeons, some two hundred are entitled to preference, either from their political influence or their following among the magnates in authority, and they are expecting to leave the others of less pretensions to take care of themselves as best they can.

If the railroad surgeon was always employed on account of merit and special fitness for his class of work, it should naturally follow that the large and strong railway companies would have the best men in the profession. But when it is known to all who care to look into the qualifications of those who are appointed, that they are selected from considerations of a different character, and that some have no claims to surgical skill, it becomes a question as to the manner in which the more eminent surgeons in railway service are to be determined by this select body.

It is evident that self-glorification is at the bottom of this movement on the part of the surgeons who met in Chicago for the purpose of getting up this special organization. All are prepared to hear an expression of supreme indifference as to the opinion of an outsider, such as myself, and of proud contempt for the feelings of those railway surgeons who are left out. But it affords the writer some satisfaction to assure these gentlemen that he has never been an applicant for any appointment in the railway service, and that the only basis upon which such a position would be accepted is that of conformity to the rates which are recognized for such service by the profession.

The feature which calls for consideration in connection with these cut-rates which are accepted by railway surgeons, is that they might insist upon regular compensation for their services and receive full pay, if all should adhere to the rules adopted by the profession in the different places where appointments are made. The medical men employed by the railway companies ought to be skillful surgeons, and the interests of all concerned will be best subserved by holding up the standard of charges for their services.

The magnates cannot afford to put inferior men on duty because of the low price at which they may be willing to accept an appointment, and it is very evident that a firm demand upon them by all who occupy positions at present, and those whose services may be sought in the future, would effect a change in this respect.

If the different associations of railway surgeons would adopt regulations requiring regular rates of compensation, the status of the members would be restored, with benefit in the long run to the railroad companies. J. M'F. G.

